# Electroacupuncture for thalidomide/bortezomib-induced peripheral neuropathy in multiple myeloma: a feasibility study

**DOI:** 10.1186/1756-8722-7-41

**Published:** 2014-05-09

**Authors:** M Kay Garcia, Lorenzo Cohen, Ying Guo, Yuhong Zhou, Bing You, Joseph Chiang, Robert Z Orlowski, Donna Weber, Jatin Shah, Raymond Alexanian, Sheeba Thomas, Jorge Romaguera, Liang Zhang, Maria Badillo, Yiming Chen, Qi Wei, Richard Lee, Kay Delasalle, Vivian Green, Michael Wang

**Affiliations:** 1 Integrative Medicine Program, The University of Texas MD Anderson Cancer Center, 1515 Holcombe Boulevard, Unit 0462, Houston, Texas 77030, USA; 2 Department of Rehabilitation Medicine, The University of Texas MD Anderson Cancer Center, 1515 Holcombe Boulevard, Unit 1414, Houston, Texas 77030, USA; 3 Department of Medical Oncology, Zhongshan Hospital, Fudan University, 180 Feng Lin Road, Shanghai, P.R. China; 4 American College of Acupuncture and Oriental Medicine, 9100 Park West Drive, Houston, Texas 77063, USA; 5 Department of Anesthesiology & Peri-Operative Medicine, The University of Texas MD Anderson Cancer Center, 1515 Holcombe Boulevard, Unit 0409, Houston, Texas 77030, USA; 6 Department of Lymphoma/Myeloma, The University of Texas MD Anderson Cancer Center, 1515 Holcombe Boulevard, Unit 0429, Houston, Texas 77030, USA; 7 Department of Experimental Therapeutics, The University of Texas MD Anderson Cancer Center, 1515 Holcombe Boulevard, Houston, Texas 77030, USA

**Keywords:** Acupuncture, Myeloma, Chemotherapy, Peripheral neuropathy, Integrative medicine

## Abstract

**Background:**

This single-arm study evaluated feasibility, safety, and initial efficacy of electroacupuncture for thalidomide/bortezomib-induced peripheral neuropathy (PN) in cancer patients with multiple myeloma.

**Methods:**

Patients with neuropathy ≥ grade 2 received 20 acupuncture treatments over 9 weeks.

**Results:**

For the 19 evaluable patients, Functional Assessment of Cancer Therapy/Gynecological Oncology Group-Neurotoxicity (FACT/GOG/NTX) mean (SD) scores improved significantly between baseline and week 13 (20.8 [9.6] vs 13.2 [8.5], p = 0.0002). Moderate effect size differences began on week 4, with the largest effect size differences found at week 9 for FACT/GOG/NTX scores, *worst* pain in the last 24 hours, and pain *severity* (Cohen’s *d* = 1.43, 1.19, and 1.08, respectively) and continuing through week 13 (Cohen’s *d* = 0.86, 0.88, and 0.90, respectively). From baseline to week 13, additional significant improvements were seen as follows: postural stability (1.0 [0.6] vs 0.8 [0.4], p = 0.02); coin test (10.0 [7.4] vs 5.6 [1.9], p < 0.0001); button test (96.1 [144.4] vs 54.9 [47.3], p < 0.0001); and walking test (21.6 [10.0] vs 17.2 [7.7], p = 0.0003). No significant changes were seen with NCS.

**Conclusions:**

Acupuncture may help patients experiencing thalidomide- or bortezomib-induced PN. Larger, randomized, clinical trials are needed.

**Trial registration:**

ClinicalTrials.gov Identifier: NCT00891618.

## Background

Peripheral neuropathy (PN) is a common side effect of treatment for patients with multiple myeloma (MM) [1,2]. PN is often chronic and can cause severe and debilitating symptoms that negatively affect patients’ quality of life [3,4]. Thalidomide and bortezomib are remarkably effective in the treatment of MM [5,6], but studies have shown that up to 75% of patients treated with thalidomide [7-9] and one third to one half of patients treated with bortezomib [3,8-10] experience PN severe enough to require dose reduction or early termination of chemotherapy.

The pathogenesis underlying both bortezomib- and thalidomide-induced PN is unclear. For bortezomib-induced PN, previous studies have reported metabolic changes due to the accumulation of bortezomib in the dorsal root ganglia cells, mitochondrial-mediated dysregulation of Ca^2+^ homeostasis, and dysregulation of neurotrophins [10]. For thalidomide-induced PN, Johnson and colleagues found the risk of developing PN can be mediated by polymorphisms in genes that are involved in repair mechanisms and inflammation in the peripheral nervous system [11]. Overall, both agents cause significant neurotoxicity. Persistent or intermittent pain, paresthesia, and hyperaesthesia are common in patients with PN, and many patients experience serious problems with gait and balance.

The current management of chemotherapy-induced PN focuses on treating its symptoms, generally with antidepressants, non-narcotic and narcotic analgesics, and anticonvulsants [1,8,9]. The efficacy of these treatment approaches is limited [1,8,9], and the side effects may be severe. In contrast, acupuncture is a safe, minimally invasive procedure with few side-effects [12-14]. Very rare, sporadic, single cases of injury have been reported [15-17] and are usually due to negligence on the part of the practitioner rather than to the treatment itself. Although studies have shown that acupuncture may be helpful for managing neuropathic pain in patients with HIV/AIDS [18,19] and diabetes [20-24], few studies [25-28] have evaluated its use in chemotherapy-induced PN.

Acupuncture analgesia involves the integration of multiple processes at different levels of the nervous system [29]. Manual acupuncture has been shown to activate Aβ-, Aδ- and C afferent fibers, and when treatment intensity is supplemented by adding an electrical current to the needles, excitation of Aβ- and part of Aδ-fibers can enhance analgesic effects [29]. After the acupuncture treatment signal ascends through the spinal ventrolateral funiculus, a complex network of brain regions, such as the nucleus raphe magnus, periaqueductal grey, locus coeruleus, arcuate nucles, preoptic area, nucleus submedius, habenular nucleus, accumbens nucleus, caudate nucleus, septal area, amygdala, and sensorymotor cortex, become involved in signal processing [29].

Inherited genetic factors also play a role in individual response to acupuncture, and multiple molecules, such as opioid peptides (μ-, δ-, and к-receptors), glutamate (NMDA and AMPA/KA receptors), 5-hydroxytryptamine, and cholecystokinin octapeptide, help mediate analgesia [29]. The release of opioid peptides evoked by electroacupuncture depends on the frequency of current delivered [30]. For example, a frequency of 2 Hz induces the gene expression of endorphins in the diencephalons, 2–15 Hz causes the release of endorphins and enkephalin in the brain and dynorphin in the spinal cord, and a frequency of 100 Hz causes the release of dynorphin in the spinal cord alone [30]. When alternating frequencies are used, these three opioids have a synergistic effect [31].

On the basis of promising findings from earlier studies [19-25,27,28,32], we designed the current trial to evaluate the feasibility, safety, and initial efficacy of acupuncture for the treatment of thalidomide- and/or bortezomib-induced chronic PN in patients with MM. Results from this study will help inform the design of a future, large randomized clinical trial.

## Results

**Figure 1:**
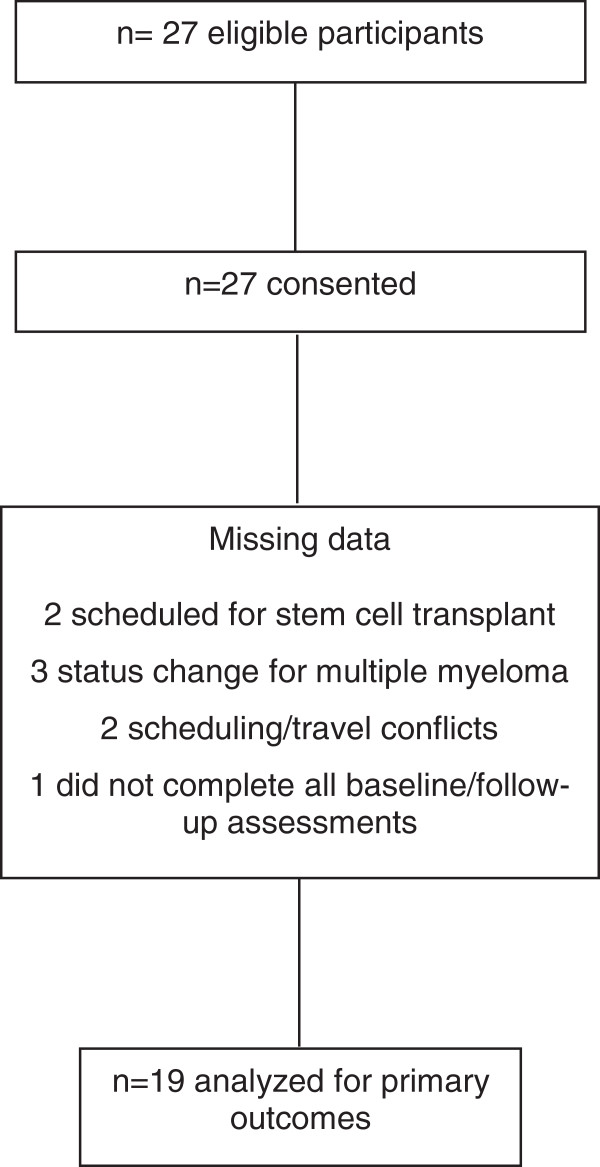
Study flow diagram.

Participant flow throughout the study is shown in Figure 1. Of 27 patients who met eligibility, all agreed to participate. Eight patients (30%) were lost to follow-up for the following reasons: disease progression (relapsed MM, n = 3), scheduled stem cell transplantation (n = 2), scheduling/travel conflicts (n = 2), and failure to complete follow-up assessments (n = 1). Nineteen patients were evaluable for study analyses. No serious adverse events related to acupuncture were reported; however, one patient did describe a worsening of peripheral neuropathy symptoms during the study.

### Patient characteristics

Patient characteristics are provided in Table 1. Fourteen men and five women participated, and the mean participant age was 64 years (range, 46–79 years). Thirteen (68%) patients had grade 2 and six (32%) patients had grade 3 PN. All patients had sensory neuropathy, and one had combined sensory and motor symptoms. All patients had symptoms in both the upper and lower extremities. Sixteen (84%) patients had been treated with bortezomib and three (16%) with thalidomide. The average number of months since the patients received their last doses of thalidomide and/or bortezomib was 9.8 (range, 0.75 to 41.5 months).

**Table 1:** Patient characteristics

**Characteristics**	
**Mean age, years (range)**	64 (range 46–79)
	**No. of patients (%)**
**Gender**	
Men	14 (74%)
Women	5 (26%)
**Diagnosis**	
Multiple myeloma	18 (95%)
Amyloidosis	1 (5%)
**Peripheral Neuropathy (PN)**	
Grade 2	13 (68%)
Grade 3	6 (32%)
**PN Treatment**	
Pregabalin	11 (58%)
Gabapentin	6 (32%)
None	2 (10%)
Pain Management*	
Hydrocodone	4 (21%)
Oxycodone	3 (16%)
Morphine	1 (5%)
Hydromorphone + bupivacaine (pump)	1 (5%)
**Prior Therapy**	
Bortezomib	16 (84%)
Thalidomide	3 (16%)
**Dose of Prior Therapy**	
Bortezomib, mg (range)	31.7 (10.5-64)
Thalidomide, g (range)	84.7 (8.4-163)
**No. Months Since Last Dose (range)**	9.8 (0.75-41.5)

*Patients had to remain on same treatment regimen; however, minor adjustments in dosage were allowed.

For neuropathy symptoms, 11 (58%) patients took pregabalin, and six (32%) took gabapentin during the study. For pain control, four (21%) patients took hydrocodone, three (16%) oxycodone, one (5%) morphine, and one (5%) hydromorphone. Two (10%) patients did not take opioids, pregabalin, or gabapentin for symptom management during the study.

### Efficacy

Patient reported outcomes (PROs) are provided in Table 2. For the Functional Assessment of Cancer Therapy/Gynecological Oncology Group-Neurotoxicity scale (FACT/GOG/NTX), there was a main effect of time (p < 0.0001). The mean (standard deviation [SD]) scores improved significantly between baseline (20.8 [9.6]) and all subsequent time points: week 4: 16.7 (9.4), p = 0.02; week 9: 9.9 (5.6), p < 0.0001; and week 13:13.2 (8.5), p = 0.0002. A moderate effect size (Table 3) was found by week 4 (Cohen’s *d =* 0.4), with the largest effect size occurring between baseline and week 9 (Cohen’s *d* = 1.4). The effect size remained large at the one-month follow-up visit at week 13 (Cohen’s *d* = 0.9).

**Table 2:** Patient-reported outcomes (PROs)

**Measure**	**Time point**	**n**	**Mean (SD)**	**p value**		**p value**
				**Paired t test**	**Non-parametric signed rank test**	Mixed model analysis*
**FACT/GOG-NTX**^**†**^	Baseline	19	20.8 (9.6)	–	–	–
	4 weeks	18	16.7 (9.4)	0.0114	0.018	0.0263
	9 weeks	15	9.9 (5.6)	0.0009	<0.0001	<0.0001
	13 weeks	15	13.2 (8.5)	0.0007	<0.0002	<0.0001
**BPI-SF**^**‡**^						
Pain	Baseline	18	25.4 (18.5)	–	–	–
*severity*	4 weeks	18	18.2 (16.4)	0.0048	0.0052	0.0056
	9 weeks	16	15.1 (14.5)	0.0004	0.0001	<0.0001
	13 weeks	16	17.6 (16.6)	0.004	0.0007	<0.0001
Pain	Baseline	18	25.4 (18.5)	–	–	–
*interference*	4 weeks	18	18.2 (16.4)	0.0048	0.0052	0.0056
	9 weeks	16	15.1 (14.5)	0.0004	0.0001	<0.0001
	13 weeks	16	17.6 (16.6)	0.004	0.0007	<0.0001
*Worst* pain in	Baseline	18	6.2 (3.5)	–	–	–
last 24 hours	4 weeks	18	3.8 (2.7)	0.0057	0.0049	0.0004
	9 weeks	16	2.9 (2.1)	<0.0001	0.0002	<0.0001
	13 weeks	15	3.6 (2.5)	<0.0001	0.0006	<0.0001
**FACT-G**^**§**^						
Physical	Baseline	18	9.2 (6.1)	–	–	–
Well-being	4 weeks	18	7.2 (5.6)	0.3	0.4	0.3
	9 weeks	14	5.0 (3.8)	<0.05	0.02	0.002
	13 weeks	16	5.5 (4.3)	0.01	0.002	0.0004
Social/family	Baseline	19	20.5 (6.3)	–	–	–
Well-being	4 weeks	16	19.7 (6.3)	0.7	0.8	0.4
	9 weeks	14	19.4 (8.5)	0.3	0.7	0.1
	13 weeks	15	19.6 (7.0)	0.3	0.6	0.3
Emotional	Baseline	19	5.3 (5.5)	–	–	–
Well-being	4 weeks	18	4.4 (4.0)	0.5	0.6	0.5
	9 weeks	16	3.8 (4.3)	0.4	0.3	0.2
	1 month	16	4.1 (3.9)	0.3	0.4	0.2
Functional	Baseline	19	20.8 (6.7)	–	–	–
Well-being	4 weeks	17	19.7 (8.4)	0.07	0.09	<0.05
	9 weeks	14	19.6 (7.0)	0.1	0.1	0.1
	1 month	15	20.4 (8.9)	0.4	0.4	0.3

*Mixed model analysis for time effect was performed for variables with four assessments.

^†^Functional Assessment of Cancer Therapy/Gynecologic Oncology Group-Neurotoxicity Scale.

^‡^Brief Pain Inventory – Short Form.

^§^Functional Assessment of Cancer Therapy – General Scale.

**Table 3:** **Cohen’s *d *** **effect size estimates**

**Measure**	**n**	**Mean (SD) Change from Baseline**	**Cohen’s *d***
FACT/GOG-NTX*			
Week 4	19	4.1 (0.2)	0.4
Week 9	19	10.9 (4.1)	1.4
Week 13	19	7.6 (1.2)	0.9
**BPI-SF**^**†**^			
*Worst pain*			
Week 4	18	2.4 (0.8)	0.8
Week 9	18	3.3 (1.4)	1.2
Week 13	18	2.6 (1.0)	0.9
*Severity*			
Week 4	18	7.1 (3.4)	0.7
Week 9	18	10.6 (4.8)	1.1
Week 13	18	9.1 (4.0)	0.9
*Interference*			
Week 4	18	7.2 (2.1)	0.4
Week 9	18	10.3 (4.0)	0.6
Week 13	18	7.8 (1.9)	0.5

*Functional Assessment of Cancer Therapy/Gynecologic Oncology Group-Neurotoxicity Scale.

^†^Brief Pain Inventory – Short Form.

For Brief Pain Inventory-Short Form (BPI-SF) scores, there was a main effect of time for all subscales (all p’s < 0.0001). Mean (SD) scores revealed significant improvements in pain *severity* and *interference* and *worst* pain in 24 hours at all time points (Table 2), with large effect size differences (Table 3) found for both pain *severity* (week 9 = 1.1 and week 13 = 0.9) and *worst* pain in 24 hours (week 4 = 0.8; week 9 = 1.2; and week 13 = 0.9). For pain *interference*, Cohen’s *d* effect size estimates were moderate (week 4 = 0.4; week 9 = 0.6, and week 13 = 0.5).

The Functional Assessment of Cancer Therapy-General (FACT-G) scores revealed significant main effect of time for the physical well-being scale (p = 0.0004) with improvement from baseline to the end of treatment at week 9 (9.2 [6.1] vs 5.0 [3.8], p < 0.05) and from baseline to follow-up at week 13 (9.2 [6.1] vs 5.5 [4.3, p = 0.01). No improvements were seen in social/family, emotional, or functional well-being (Table 2).

Timed-function test scores improved from baseline to one-month follow-up as follows: coin test (10.0 [7.4] vs 5.6 [1.9], p < 0.0001); button test (96.1 [144.4] vs 54.9 [47.3], p < 0.0001); walking test (21.6 [10.0] vs 17.2 [7.7], p = 0.0003); and postural stability (1.0 [0.6] vs 0.8 [0.4], p = 0.02). Marginal improvement was seen in fall risk, but it was not statistically significant (Table 4). No significant changes were found for the nerve conduction studies (NCS data not shown).

**Table 4:** Timed function tests

**Measure**	**Time point**	**n**	**Mean (SD)**	**p value**	
				**Paired t test**	**Non-parametric signed rank test**
Timed function tests*					
Walk test	Baseline	19	21.6 (10)	–	–
	13 weeks	19	17.2 (7.7)	0.0005	0.0003
Button test	Baseline	19	96.1 (144.4)	–	–
	13 weeks	19	54.9 (47.3)	0.16	<0.0001
Coin test	Baseline	19	10.0 (7.4)	–	–
	13 weeks	19	5.6 (1.9)	0.0123	<0.0001
**Balance tests**					
Postural stability	Baseline	19	1.0 (0.6)	–	–
	13 weeks	19	0.8 (0.4)	0.06	0.02
Fall risk	Baseline	18	1.9 (0.8)	–	–
	13 weeks	18	1.6 (0.6)	0.06	0.07

*In seconds.

## Discussion

With an extended treatment period lasting 9 weeks and involving 20 acupuncture sessions at two to three times per week, our criteria for feasibility were that at least half of the patients approached would agree to participate and that the drop-out rate would be less than one third. In this single-arm trial, all patients who were referred to the research nurse and determined to be eligible agreed to participate, and the drop-out rate was 30% (8/27); thus, the acupuncture service provided was feasible.

In terms of determining initial efficacy, the PRO scores indicated significant improvement in PN symptoms starting at week 4 (Cohen’s d = 0.4; p = 0.02), with the largest improvement seen at the end of treatment at week 9 (Cohen’s *d* = 1.4; p <0.0001) and remaining through the one-month follow-up at week 13 (Cohen’s *d* = 0.9; p = 0.0002). Additionally, significant improvement was found for pain *severity* and *interference* and for *worst* pain experienced over 24 hours (see Tables 2 and 3). Again, these changes were seen by week 4 and persisted at the one-month follow-up.

Although this study will help inform future trials, there were several limitations. As a feasibility study, it was limited by a small sample size and lack of a comparison group. According to the United States Food and Drug Administration, however, assessment of “pain at its *worst* in the last 24 hours” on the BPI-SF is sufficient for measuring a pain reduction treatment effect [33], and a reduction of 2 points or more on a 0–10 numeric rating scale for “*worst* pain” in the last 24 hours implies a clinically significant change. A greater than 2 point mean reduction in pain from baseline was noted at each time point.

Another limitation was the inability to separate specific versus non-specific placebo effects of the acupuncture treatment. Clearly, there is a strong placebo component of acupuncture [34-36], and future sham-controlled trials are needed to parse-out various treatment elements. With this trial design, we cannot rule out the possibility of a placebo effect; however, participants had persistent PN in spite of having had previous treatment using standard of care methods, with which there would also have been expected benefit due to placebo. Finally, this study was limited by the fact that there was no long-term follow-up, and the extent to which symptoms returned is unknown. Future trials may need to include long-term treatment for maintenance of symptom relief.

## Conclusions

Controlling chemotherapy-induced PN while managing an underlying hematologic malignancy presents many challenges [1]. Symptoms may be severe enough to require dose reduction or termination of chemotherapy. Often, symptoms become chronic and negatively impact patients’ quality of life [3,4]. In this small, non-randomized feasibility study, reductions in BPI-SF pain *severity* and *interference* as well as improvements in FACT/GOG/NTX scores and improvements in objective timed function tests provide compelling evidence that acupuncture may be of benefit as an adjunct to symptom management in chemotherapy-induced PN. Due to the dearth of literature in this area and the chronicity of debilitating symptoms, further investigation is warranted. Randomized, controlled trials with strong methodology to reduce risk of bias and parse-out specific versus non-specific effects are greatly needed.

## Methods

### Study population

Prior to activating this non-randomized pilot study, we obtained approval from the MD Anderson Cancer Center Institutional Review Board. Potential participants for this study were identified by faculty in the Department of Lymphoma/Myeloma at The University of Texas MD Anderson Cancer Center in Houston, Texas, and referred to the research nurse for assessment of eligibility and to obtain informed consent. Eligible participants were more than 18 years of age and had a diagnosis of MM. They had grade 2 or higher neuropathy according to the Common Terminology Criteria for Adverse Events, v3.0 [37] in spite of previous treatment with gabapentin, duloxetin, and/or pregabalin. Participants had to remain on the same medications throughout the study period; however, minor adjustments in dosage were allowed. An Eastern Co-operative Oncology Group Performance Status of ≤ 2 was required for study inclusion.

Patients were excluded if they were currently being treated with thalidomide and/or bortezomib or if they had a local infection or deformities at or near the acupuncture sites. Additionally, patients were excluded for any of the following reasons: using alternative medicines that could affect PN symptoms, such as herbal agents, or taking high-dose vitamins; having known coagulopathy or taking heparin or warfarin; having platelets < 50000/μL or a white blood cell count < 3000/μL; having active central nervous system disease, a pacemaker, mental incapacitation or a significant emotional or psychological disorder that could interfere with study participation; being pregnant or lactating; having a history of chronic alcohol misuse or diabetes- or HIV-related PN; having prior acupuncture treatment for any indication within 30 days of enrollment; and receiving active treatment for MM.

### Study design and treatment

The treatment methods (i.e., point selection, type of stimulation, needling techniques, and treatment schedule) were chosen by a panel of expert faculty from the American College of Acupuncture and Oriental Medicine in Houston. Patients received acupuncture three times per week for 4 weeks followed by 1 week off (week 5) and then twice per week for 4 more weeks (i.e., 20 treatments over 9 weeks). Two licensed staff acupuncturists with over 30 years combined experience, and who were trained in the same masters’ degree program, provided all treatments.

Standardized techniques for locating points and depth of insertion were utilized [38,39] while patients were treated in a comfortable supine position. All treatments were given using needles (36–38 gauge and 30 mm length) manufactured by Seirin Corporation (Shizuoka, Japan). Bilateral upper and lower extremities were treated. The specific points selected for this trial were LI4, SI3, Baxie 2, Baxie 3, Lv3, Sp6, Gb42, St 36, Bafeng 2, Bafeng 3, Du 20, CV4, and CV6 (see Table 5). After *de qi* (a sensation of numbness, tingling, or warmth at the needle insertion site) was experienced by the patient, electrical stimulation was applied bilaterally as follows: from LI4 (negative) to SI3 (positive) and from Lv3 (negative) to Gb42 (positive) at 2–100 Hz for 20 minutes. All needles were then removed.

**Table 5:** Acupuncture treatment

**Points**	**Sides**	**Depth**	**Angle**	**Gauge**
Lv 3*	X2	0.5-1 in.	perpendicular	36
Sp 6	X2	0.8-1 in.	perpendicular	36
Gb 42*	X2	0.5-1 in.	perpendicular	36
St 36	X2	1-1.5 in.	perpendicular	36
LI 4*	X2	0.5-1 in.	perpendicular	36
SI 3*	X2	0.5-1 in.	perpendicular	36
CV 4	midline	0.5-1 in.	perpendicular	36
CV 6	midline	0.5-1 in.	perpendicular	36
Du 20 (posterior direction against channel flow)	midline	0.8-1 in.	perpendicular	36
Baxie 2 & 3 (between 2nd & 3rd and 3rd & 4th fingers)^†^	X2	0.5-1 in.	Horizontal (towards the wrist)	36
Bafeng 2 & 3 (between 2nd & 3rd and 3rd & 4th toes)^†^	X2	0.5-1 in.	Oblique (towards the heel)	36

*After *de qi* was detected, electrical stimulation was applied using an electro-acupuncture stimulator (IC-1107, ITO Co., LTD, Tokyo, Japan) at alternating frequencies (2–100 Hz) as follows: pair one-Lv3 negative, Gb 42 positive; pair two-LI 4 negative, SI 3 positive. Voltage was adjusted to patient tolerance (ie., able to feel strong stimulation without pain).

^†^Thumb and great toe are considered 1st digit.

### Assessments

To determine feasibility and safety, we tracked subject recruitment, attrition, compliance with treatment and follow-up regimens, and adverse events. Our criteria for success in terms of feasibility were that that at least 50% of the patients approached would agree to participate and fewer than 33% of the patients who gave consent to participate would drop out after recruitment.

For efficacy, the FACT/GOG/NTX scale was used to assess the primary endpoint for PN, and patients had to complete at least 80% of the treatment sessions to be evaluable. The FACT-G survey and the Brief Pain Inventory-Short Form (BPI-SF) were also used to evaluate PROs. For objective outcomes, timed function tests (i.e., the coin test, button test, walking test, and postural stability/fall risk) and NCS were performed. The NCS consisted of bilateral radial and sural sensory nerve conduction and bilateral tibial motor nerve conduction tests. We used previously established age-adjusted values to categorize sensory and motor nerve action potential amplitudes as normal or abnormal [40].

### Statistical considerations

Patient characteristics were assessed by frequency distributions, means, and ranges for relevant variables. For PROs, mean values/scores were calculated at baseline and weeks 4, 9, and 13 and compared for statistical significance. For variables with four time points, mixed model analyses were performed for time effect. Changes in function tests and NCS were compared from baseline to week 13. All comparisons were made using the paired Student’s *t*-test as well as the non-parametric signed rank test. Finally, in order to determine the strength of change due to the intervention, Cohen’s *d* effect size estimates were calculated for both the pain and neuropathy scores. Statistical analyses were performed using SPSS, v20 statistical software (IBM Corporation, Armonk, NY).

## Competing interests

The authors declare that they have no competing interests.

## Authors’ contributions

All authors participated in approval of the final manuscript. MW obtained funding and was responsible for overseeing all aspects of the study. MG, LC, YG, MB, YC, and QW participated in data collection, management, analysis, and interpretation. ZY, YB, JC, RO, DW, JS, RA, ST, JR, RL, LZ, KD, and VG provided expertise during study development, design, data collection, interpretation, and writing of the manuscript.

## Authors’ information

M. Kay Garcia and Lorenzo Cohen are co-first authors.
